# Total Glucosides of Paeony Capsule Plus Compound Glycyrrhizin Tablets for the Treatment of Severe Alopecia Areata in Children: A Randomized Controlled Trial

**DOI:** 10.1155/2013/378219

**Published:** 2013-09-24

**Authors:** Dingquan Yang, Jingwen Zheng, Yuming Zhang, Yueli Jin, Chaonan Gan, Yanping Bai

**Affiliations:** Department of Dermatology, China-Japan Friendship Hospital Affiliated to Ministry of Public Health, Beijing 100029, China

## Abstract

Total glucosides of paeony capsule (TGPC) and compound glycyrrhizin tablets (CGT) are plant extracts of glycosides. We conducted this study to examine the efficacy and safety of TGPC plus CGT for severe alopecia areata in children. 117 subjects were randomly allocated into TGPC plus CGT group or CGT group. For consecutive 12 months, subjects were given oral TGPC and CGT or oral CGT alone. The outcome measures included score of alopecia areata severity, effective rate, and adverse events observed in the 3rd, 6th, and 12th month. We found that the scores of alopecia areata severity of both groups were significantly reduced, and the scores of treatment group were lower than those of control group; for effective rate, there was no statistical difference between the two groups in the 3rd month, while in the 6th and 12th months the treatment group was superior compared with control group; the incidence rate of adverse events between the two groups was not statistically different, and no severe adverse events were observed. In conclusion, TGPC plus CGT appears effective and safe for severe alopecia areata in children.

## 1. Introduction

Alopecia areata in children is a specific type of alopecia areata. The mechanism of the disease is related to genetics, autoimmune, mental factors, EB virus infection, and lack of trace elements [1]. The prognosis of severe alopecia areata in children is often poor because it is not a self-limited disease, and the majority of children with alopecia totalis or alopecia universalis do not comply with a long-term treatment since their parents worry about the adverse reactions of medications [2].

Compound glycyrrhizin tablets (CGT) and total glucosides of paeony capsule (TGPC) are both plant extracts of glycosides, with the function of immunoregulation. In one of our previous studies comparing CGT and TGPC for mild and moderate alopecia areata [3], we found that both CGT and TGPC can significantly improve the treatment of alopecia areata and that there was no statistical difference between CGT and TGPC groups. Since CGT and TGPC can both regulate T cells activation and they have different targets during the T cells activation [3], we proposed that the combination of CGT and TGPC could be superior compared with CGT or TGPC alone in the treatment of alopecia areata. Clinical studies reported in China also show that CGT plus TGPC is effective in treating inflammatory skin diseases related to T cells, such as psoriasis, leucoderma, and lichen planus. 

We conducted this randomized controlled trial between August 2009 and July 2011 comparing TGPC plus CGT with CGT alone, to assess the efficacy and safety of TGPC plus CGT for the treatment of severe alopecia areata in children.

## 2. Material and Methods

### 2.1. Subjects

Children aged between 2 and 14 years, with alopecia areata severity ≥S_3_, were included in this study.

#### 2.1.1. Diagnostic Criteria [4]

Loss of hair suddenly occurres in the scalp with sharply defined areas or even all area of the scalp. Skin of the areas is smooth, without inflammation. In active stage, the hair tends to fall out on the side of the hair loss area and is likely to be pulled out (Gently pulling test proves positive). In alopecia areata trichoscopy, the hairs become narrower along the length of the strand closer to the base, presenting a characteristic “exclamation point” appearance. Concomitant symptoms are loss of the scalp, beard, bald patches of body hair, and changes of nails or even loss of any hair of the body. 

#### 2.1.2. Standard of Alopecia Areata Severity [5]

The area of hair loss was evaluated according to severity of alopecia tool (SALT): S_0_ is no hair loss; S_1_ is that the area of hair loss is less than 25%; S_2_ is that the area of hair loss is 25%~49%; S_3_ is that the area of hair loss is 50%~74%; S_4_ is that the area of hair loss is 75%~99%; S_5_B_0_ is alopecia totalis (AT), which is total scalp hair loss; S_5_B_1_ is complete scalp hair loss with partial body hair loss; S_5_B_2_ is alopecia universalis (AU), which is complete scalp hair loss and body hair loss. S_1_ and S_2_ are mild alopecia areata, and S_3_ to S_5_B_2_ are severe alopecia areata.

#### 2.1.3. Inclusion Criteria

(1) Aged between 2 and 14 years, with alopecia areata severity ≥S_3_; (2) adhered to the followup and medication protocol and obtained informed consent; (3) no previous local or systematic treatment within 4 weeks, and no local injection or general application of long-term steroid hormone treatment; (4) no severe liver and kidney dysfunction or fluid and electrolyte disorders.

#### 2.1.4. Exclusion Criteria

Patients were excluded if they were allergic to TGPC or CGT and if they had congenital hypotrichosis, congenital hairless, false alopecia areata, alopecia syphilitica, trichotillomania, or alopecia cicatrisata.

#### 2.1.5. Suspension and Withdrawal Criteria

(1) Serious adverse reaction occurred; (2) protocol deviations or violations; (3) loss to followup (including withdrawal by themselves); (4) withdrawal of the informed content; (5) clearly allergic to TGPC or CGT.

### 2.2. Interventions

Both groups were given 5 mg of vitamin B_2_, twice daily. Patients in treatment group were given oral TGPC (manufactured by Ningbo Lihua Pharmaceutical Co., Ltd., H20055058, 300 mg/capsule), 3 times per day and 300 mg per time, and CGT (manufactured by Japanese Minophagen Pharmaceutical Co., Ltd., J20080080, 25 mg/tablet), 3 times per day and 25 mg per time. Patients in control group were only given CGT, and the administration was the same as the treatment group. The duration of treatment of both groups was 12 months. 

### 2.3. Outcome Measures

Participants were advised to see the doctor once each month. The changes in score of alopecia areata severity, effective rate, and adverse events of each group were observed in the 3rd month, 6th month, and 12th month after treatment, respectively. The blood, function of liver and kidney, and electrolyte were tested before treatment and in the 3rd month, 6th month, and 12th month after treatment, respectively. The safety was evaluated in the 12th month according to the incidence rate of adverse events.

#### 2.3.1. Severity of Alopecia Areata Assessment

S_0_ was given 0 score; S_1_ was given 1 score; S_2_ was given 2 scores; S_3_ was given 3 scores; S_4_ was given 4 scores; S_5_ was given 5 scores; S_5_B_0_ was given 6 scores; S_5_B_1_ was given 7 scores; S_5_B_2_ was given 8 scores. The improvement of the area of alopecia areata was assessed according to the changed scores of the two groups.

#### 2.3.2. Effective Rate Criteria

According to the criteria of Weiss et al. [6], there were four grades: cured, markedly effective, effective, and ineffective. Cured: all hairs grew out again, normal in density of distribution, color and luster, and negative in pulling hair test; markedly effective: 50% of hairs grew out again, almost normal in density of distribution, color and luster, with many fine hair turning into hair, and negative in pulling hair test; effective: 10% of hairs grew out again (including fine hair) but grew slowly, and negative or positive in pulling hair test; ineffective: after a treatment of more than 3 months, no new hairs grew out or new hairs just less than 10% or continued with hair loss. Effective rate = (cured cases + markedly effective cases)/total cases × 100%.

#### 2.3.3. Adverse Events

We recorded the adverse reactions during the study. The TGPC would possibly cause abdominal pain, loose stool, and increase in stool frequency. The CGT would possibly cause hypokalemia, increase in blood pressure and weight, edema, decrease in muscle strength, and muscle pain. We counted the number of adverse events and calculated the incidence rate of adverse event with the following formula: (cases of adverse event/total cases) × 100%.

### 2.4. Compliance Strategy

Since parents play an important role in the treatment of alopecia areata in children, we tried our best to have a good communication with the parents or guardians of all children participants and conducted safety monitor of the prescribed medications, in order to keep good compliance of the treatment.

### 2.5. Statistical Analysis

All data were input into computer and managed by dBASE database. Data analyses were performed by SPSS16.0. The *X* ^2^ test was used for enumeration data, *t* test was used for measurement data, and Mann-Whitney test was used for ranked data. *P* < 0.05 was considered as statistically significant. We set up the significant figure to an accuracy of 0.01.

## 3. Results

### 3.1. Baseline Information

A total of 117 cases of eligible outpatients from China-Japan Friendship Hospital were randomly divided into treatment group (*n* = 60) or control group (*n* = 57) according to a random number table. In treatment group, there were 36 males and 24 females, aged between 2 and 14 years, and the participants suffered from alopecia areata for 6 months to 12 years, with 12 cases of S_3_, 16 S_4_, 18 S_5_, and 14 S_5_B_2_. In control group, there were 32 males and 25 females, aged between 2 and 14 years, and the participants suffered from alopecia areata for 9 months to 11 years, with 9 cases of S_3_, 12 S_4_, 20 S_5_, and 16 S_5_B_2_. There was no statistical difference between the two groups in sex ratio, age, duration of alopecia areata, and severity of alopecia areata (*P* > 0.05). Informed content was obtained from each participant.

### 3.2. Changes in Scores of Alopecia Areata Severity before and after Treatment

There was no statistical difference between groups in alopecia areata severity before treatment (*P* > 0.05), which was comparable. The alopecia areata severity scores in both groups became lower with the duration of treatment, and there was statistical difference within each group before and after treatment (*P* < 0.01), which demonstrated that both TGC and CGT were effective and alopecia areata severity was gradually reduced with the duration of treatment. In the 3rd, 6th, and 12th months after treatment, the scores of alopecia areata severity of the treatment group were much lower than those of control group, and there was statistical difference between the two groups (*P* < 0.05), which demonstrated that the treatment group was superior in the improvement of alopecia areata compared with control group (Table 1).

### 3.3. Effective Rate

In the 3rd month after treatment, there was no statistical difference between the two groups (*P* > 0.05), while in the 6th and 12ths month after treatment, the difference was statistically significant (*P* < 0.05), and the treatment group was superior to control group, which showed that the CGT plus TGPC was superior to CGT alone in the treatment of severe alopecia areata in children (Table 2).

### 3.4. Adverse Events

There were 7 cases of adverse events in treatment group and 6 cases in control group. All adverse events were mild and released or disappeared gradually after decreasing the amount of medication. There was no statistically different in the incidence rate of adverse events between the two groups (*P* > 0.05) (Table 3).

## 4. Discussion

The morbidity of alopecia areata in population is 0.1-0.2% [7], and the morbidity of alopecia areata for each person is 2%. Alopecia areata can attack people of any ethnic group of all ages [8], especially children under 3 years. For adolescenence in China, the average age of incidence is 10 and the ratio of male and female is 1.4 : 1, and boys with alopecia areata in early and middle stage are much severer [9]. Severe alopecia areata in children often has poor prognosis. The loss of hair has great influence on physical appearance and psychology, which severely impacts the quality of life of both children and their family. They often suffer from severe emotional blow. Current treatments like glucocorticoid, immunosuppressor, and immune stimulus are hard to be accepted due to their adverse reactions. So, parents and children turn to traditional Chinese medicine or other complementary and alternative medicine for treatment. However, because of the complex treatment procedure of traditional Chinese medicine, children also cannot comply with the treatment for a long time. Therefore, effective and safe treatment methods are urgently needed for the treatment of severe alopecia areata in children. 

The cause and mechanism of alopecia areata is unclear yet. It is thought to be possibly associated to autoimmune diseases, emotional stress, and heredity. In the normal hair growth phase, the hair bulbs have characteristics of immune privilege, and low expression level of major histocompatibility antigen MHC-1*α*, which block the immune T cells CD8^+^ to recognize antigen [10]. The immune mechanism of alopecia areata is reflected in the release of autoantigen from melanocytes, removal of immune privilege, and the abnormal CD4^+^ and CD25^+^ T cells. The specific model of immune response is CD8^+^ T cells as major activator and CD4^+^ T cells as assistant, mediating immune response mainly with cytokines Th1 [11]. There are two steps during the immune response: (1) CD8^+^ cytotoxic T cells recognize the MRP (melanocyte related protein, generated only in growth phase), presented by melanocytes and/or keratinocyte MHC-I; (2) the autoantigen of melanocytes and keratinocyte, destroyed by CD8^+^ cytotoxic T cells, is presented to CD4^+^ T cells by MHC-II, resulting in a series of response to destroy hair follicles and tissues around it [12]. Emotional stress can aggravate alopecia areata through affecting inflammatory reaction. Upon stimulation of acute and repeated emotional stress, the release of vasopressin mRNA from hypothalamus and corticosteroids mRNA from pituitary is remarkably increasing, which indicates the relationship between alopecia areata and emotional stress [13]. The genetic factors reflect in family aggregation. 20% adult alopecia areata patients and 8%–52% children with alopecia areata have family members with alopecia areata, and for one identical twin if alopecia areata occurs, 55% of them will both have alopecia areata [14]. Further research is needed to explore the mechanism.

Childhood is the critical period of physical and mental development. Though alopecia areata is not a lethal or disabling disorder, it can have great influence on the psychology of children, which may result in psychological disorders such as self-abasement, lack of confidence, and autism. At present, the main treatments of alopecia areata in children are external medication and physical therapy. The conventional drugs are glucocorticoid, Minoxidil, immune stimulus, and calcineurin inhibitor, and the physical therapies mainly include ultraviolet A (UVA) and ultraviolet B (UVB) [15]. Because these methods have a risk of sensitization caused by local stimulation, systematic absorption, and potential carcinogenic factor, patients are difficult to comply with long-term treatment. According to our study, CGT (CGT) and total glucosides of paeony capsule (TGPC) are plant extracts of glycosides, with the function of anti-inflammation and immunoregulation, and can effectively treat alopecia areata. Meanwhile, they are safe and convenient in the treatment of alopecia areata in children.

CGT have anti-inflammatory, antiallergic, steroid, anticomplementary activity, and immunoregulation effects. It is commonly used to treat mild to moderate alopecia areata, due to the function of inhibiting the CD4^+^ and CD8^+^ T cells and the cytokine generated by CD4^+^ and CD8^+^ T cells [16], with good efficacy [17]. The main active ingredient of White Paeony is a group of glycosides substance, collectively called total glucosides of paeony (TGP). It can inhibit and regulate immune response, be anti-inflammatory, relieve pain, and protect the liver [18]. The mechanism of its immunoregulation may be the inhibition of antigen presentation to inhibit the cytokine induced by T cells, downregulate the expression of TNF-*α* and IL-8 [19], and adjust the imbalance of peripheral blood T-cell subsets [20], and meanwhile it can inhibit the generation of specific antibody by B lymphocyte. Therefore, it plays a role of bidirectional regulation from the beginning stage of immune response to adjust the immune disorder, including action on multiple stages of the immune response [21]. The main mechanism of TGP for the treatment of alopecia areata may be the inhibition of T lymphocyte from the beginning stage of immune response and cytokine generated by T cells.

## 5. Conclusion

This study demonstrated that TGPC plus CGT and CGT alone can lower the alopecia areata severity, and both groups had good clinical efficacy for the treatment of severe alopecia areata in children. In the first three months of treatment, the efficacy of the two groups was similar, while in the 6th and 12th months after treatment the treatment group was superior to control group. No adverse events were observed during the consecutive 12 months of treatment. All adverse events were mild. This study showed that TGPC plus CGT and CGT alone are effective and safe in the treatment of severe alopecia areata in children, and the combination treatment is more effective, which can be recommended for clinical application. 

## Figures and Tables

**Table 1:** Changes in scores of alopecia areata severity of severe alopecia areata in children before and after treatment.

Group	Case	Before treatment	3rd month after treatment	6th month after treatment	12th month after treatment
Treatment	60	6.4 ± 1.1	4.9 ± 1.6^△^	3.1 ± 1.2^△^	1.5 ± 0.9^△^
Control	57	6.6 ± 1.3*	5.5 ± 1.4^△▲^	4.1 ± 1.7^△▲^	2.4 ± 1.3^△▲^

*P > 0.05; ^△^ *P* < 0.01; ^▲^ *P* < 0.05.

**Table 2:** Comparison of the effective rate between groups for severe alopecia areata in children.

Group	Case	Duration	Cured	Markedly effective	Effective	Ineffective	Effective rate (%)
Treatment	60	3rd month	2	25	21	12	45.00
		6th month	6	30	18	6	60.00
		12th month	13	36	9	2	81.67
Control	57	3rd month*	0	20	23	14	35.09
		6th month^▲^	2	23	24	8	43.86
		12th month^▲^	5	26	20	6	54.39

*P > 0.05; ^▲^ *P* < 0.05 (*P* value was 0.248, 0.047, and 0.001, resp.; *Z* value was 1.155, 1.998, and 3.303, resp.); effective rate = (cured cases + markedly effective cases)/total cases × 100%.

**Table 3:** Comparison of the adverse events of two groups.

Group	Case	Adverse event	Abdominal pain	Loose stool	Increase in stool frequency	Hypokalemia	Rash	Edema	Weight gain	Decrease in muscle strength	Incidence rate (%)
Treatment	60	7	2	5	4	1	—	1	—	1	11.67
Control	57	6*	—	—	—	1	2	3	2	1	10.53

*P > 0.05.
